# Pre-exposure prophylaxis for HIV prevention preferences among young adult African American men who have sex with men

**DOI:** 10.1371/journal.pone.0209484

**Published:** 2018-12-28

**Authors:** Rupa R. Patel, John S. Crane, Julia López, Philip A. Chan, Albert Y. Liu, Rubabin Tooba, Aimee S. James

**Affiliations:** 1 Division of Infectious Diseases, Washington University in St. Louis, St. Louis, Missouri, United States of America; 2 Department of Obstetrics and Gynecology, Washington University in St. Louis, St. Louis, Missouri, United States of America; 3 Division of Infectious Diseases, Brown University, Providence, Rhode Island, United States of America; 4 Department of Medicine, University of California San Francisco, San Francisco, California, United States of America; 5 Bridge HIV, San Francisco Department of Public Health, San Francisco, California, United States of America; 6 Washington University School of Medicine, Washington University in St. Louis, St. Louis, Missouri, United States of America; 7 Division of Public Health Sciences, Washington University in St. Louis, St. Louis, Missouri, United States of America; Sefako Makgatho Health Sciences University, SOUTH AFRICA

## Abstract

Pre-exposure prophylaxis (PrEP) is effective in preventing HIV infections among men who have sex with men (MSM). PrEP uptake and adherence remain low and product preferences are unknown, especially among young African American MSM who are most at-risk. We conducted 26 qualitative interviews from 2014–2016 among young adult HIV-negative African American MSM regarding PrEP product preferences in Missouri. While the pill and injectable were most liked of all modalities, about a quarter preferred rectal products or patches. Most participants preferred a long-acting injectable (LAI) to daily oral pills due to better medication adherence and a dislike for taking pills. Many participants preferred daily oral pills to on-demand oral PrEP due to the inability to predict sex and the perception that insufficient time or medication would not achieve HIV protection with on-demand. A fear of needles and the perception that there would not be therapeutic levels for a long duration were concerns with injectable PrEP. Study findings highlight the need for a range of prevention options for African American MSM and can inform PrEP product development as well as dissemination and implementation efforts.

## Introduction

In the United States (US), men who have sex with men (MSM) account for 70% of all new HIV infections [1]. Young African American MSM are the most disproportionately affected MSM subpopulation accounting for 39% of the new HIV infections in the US annually [1]. At the current rates, the Centers for Disease Control and Prevention (CDC) project one in two African American MSM are at risk of acquiring HIV in their lifetime [2]. New approaches to halting the HIV epidemic among young African American MSM are needed.

Emtricitabine/tenofovir as pre-exposure prophylaxis (PrEP) is a daily oral medication that is well over 90% effective at preventing HIV infections [3–6], and holds promise to reduce infections within this population. A quarter of the MSM residing in the US are eligible for PrEP [7], but PrEP awareness and uptake has been slow [8–10], particularly among African American MSM [7, 11–15]. Adherence to daily oral PrEP among African American MSM remains a concern [16, 17]. Research on newer PrEP products is ongoing; understanding biomedical HIV prevention preferences among at-risk individuals remains a priority in order to meet the needs of the individuals who may benefit most from them [18, 19]. Two areas of PrEP product preferences to investigate are modality of PrEP (e.g., injections or patches) and the frequency of taking PrEP (e.g., daily or monthly). New PrEP products (e.g., injections, saline solutions, rectal gels, thin-film polymers, or implantable devices) and frequency of dosing (e.g., on-demand, daily, daily but episodic use, monthly, or longer periods than monthly) offer options that may enhance uptake and adherence among this population [19–26]. Previous PrEP research explored new PrEP modalities and non-daily dosing schedules, and their preferences among MSM PrEP users [27–29].

Currently, the long-acting injection (LAI), Cabotegravir (GSK1265744), used as PrEP is in phase 2b/3 efficacy clinical trials for MSM and transgender women who have sex with men [30]. Pharmacokinetic results suggest Cabotegravir as LAI PrEP can be administered every two months [31, 32]. LAI PrEP was acceptable by 79.2% of young MSM of color in a study conducted in New York City [33]. MSM in that study preferred LAI PrEP (every three months) to an oral daily pill or no PrEP treatment [33], but no other PrEP dosing schedules were investigated. Regarding the frequency of a LAI, 53.6% preferred an injection every three months to 43.2% preferring one every month [34]. John et al. reports 30.8% of 104 gay and bisexual men residing in New York preferred LAI to daily oral PrEP [35].

In addition to studying mode of administration, studies have evaluated different dosing regimens for oral PrEP, such as using non-daily PrEP dosing schedules (e.g., on-demand) [27, 28, 36–38]. The IPERGAY study found on-demand dosing effective for HIV prevention among MSM who took PrEP two pills 2 to 24 hours before sex, one pill 24 hours after first dose, and another pill 24 hours after that [28, 38]. While non-daily dosing is not recommended by the CDC, it is recommended in other countries [15, 39–43]. In an online survey of 3,217 MSM, on-demand, also referred to as event-driven, PrEP was preferred when men only had planned sex, and daily PrEP was preferred when men had frequent condomless anal sex [44]. Non-daily dosing was highly acceptable, with 75.8% of MSM reporting that they were willing to use event-driven PrEP, in comparison to daily PrEP (49.8%) [44].

Before biomedical PrEP products go on the market, modality and frequency preferences and acceptability must be addressed [24, 45, 46]. An online survey of 1,106 young MSM examined their likelihood of using topical (i.e., gels and suppositories) and systemic (i.e., pills) PrEP modalities, either daily or on-demand [47]. Most participants preferred on-demand pills (62.0%) and penile gels before sex (58.0%) to the daily oral pill (51.0%) or periodic injection (i.e., every 1–3 months) (52.7%). However African American MSM reported a higher likelihood of using daily oral pills, on-demand gels (i.e., penile and rectal), and suppositories before sex compared to White MSM [47]. Another online study of 512 MSM found daily oral pills (35.5%) and non-visible implants (34.3%) were preferred over two month injections (25.2%) and visible implants (4.3%) [48]. Participants described their modality decision based on convenience, length of protection, and privacy. These findings suggest differences in LAI and daily pill PrEP preferences among MSM.

Prior study findings regarding wide-ranging HIV prevention biomedical product acceptability suggest that there should be an array of PrEP dosing and product options, as long as there is proven efficacy with each option, in order to meet the needs of different populations [18, 34, 44, 47–49]. This qualitative study among young adult African American MSM in St. Louis, Missouri, explored: 1) PrEP modality preferences (e.g., injection or patch), 2) acceptability of and concerns with use for on-demand and LAI PrEP, and 3) preferences for oral daily PrEP in comparison to on-demand or LAI PrEP.

## Methods

### Sampling and recruitment

We conducted 26 qualitative in-depth interviews from September 2014 to February 2016 in St. Louis, Missouri. Study eligibility included being self-identified as MSM, age 18–35 years, African American, self-reported HIV-negative, reported having anal sex within the past six months, and able to give informed consent. Participants were recruited using flyers placed at designated primary care and HIV specialty clinics, community-based organization facilities and events, clubs, bars, coffee shops, restaurants, bathhouses, websites, and smartphone applications (i.e., Black Gay Chat, Grindr, Manhunt, and Jack’d). Recruitment stopped when qualitative data saturation was attained for the main themes. Study compensation included a $25 grocery card; public transportation passes were offered if needed. The study was approved by the Washington University in St. Louis Institutional Review Board (Protocol # 201407031).

### Data collection and analysis

Participants completed a self-administered, written quantitative survey before the interview. The survey included demographics (e.g., age, race, gender, education level, and annual income), sexual risk behaviors, self-perceived HIV risk, and prior PrEP use. Participants who identified as African American and at least one other race were considered multiracial. To capture sexual risk behavior, participants were asked, in the past six months, to report the number of condomless receptive anal sex partners and the number of times they had anal sex after taking alcohol or recreational drugs. A five-point Likert scale was used to assess perceived likelihood of ever becoming HIV infected. “Very unlikely” and “unlikely” were categorized as low perceived HIV risk [50]. “Somewhat likely”, “likely”, and “very likely” were categorized as moderate/high perceived HIV risk [50]. Participants were asked if they had ever heard of PrEP before the day of the survey and if they were currently taking PrEP at the time of the survey, as well as the duration. Using the same Likert scale above, participants were asked how likely they would be able to take one pill everyday.

Qualitative interviews were conducted in person, utilized a semi-structured interview guide, and were audio-recorded. Two trained, qualitative interviewers conducted the interviews. The interview guide included questions on PrEP modality preferences and reasons for that preference. PrEP in the form of a pill, an injection, rectal gel/lubricant or enema, skin patch or other preferred form were specifically asked about; respondents could mention their preference for more than one modality. We asked about preferences for PrEP dosing frequencies. Questions regarding frequency of dosing were followed up by probing on specific timing options (i.e., daily, every week, every month, every three months, or another time interval). We assessed on-demand oral and LAI PrEP acceptability, defined as willing to use, and concerns with use of both [27, 30, 32, 38]. On-demand PrEP was explained in the interviews as “using two pills before sex and two pills after sex.” LAI was explained in the interviews as “an injection that could be given every three months” [30, 32]. We also directly asked interviewees their preferences for daily oral PrEP compared to on-demand or LAI PrEP [27, 30, 32, 38].

Audio data were transcribed and de-identified. Codes were inductively developed using a grounded theory approach and a codebook was created. The transcripts were double-coded using NVivo software [51]. The emerging themes were organized into categories for analysis. Responses to questions regarding acceptability were categorized as “acceptable” and “not acceptable.” Responses to the direct comparisons for daily oral and on-demand PrEP were categorized as “daily,” on-demand”, or “indifferent.” Participants were coded as preferring oral daily PrEP to on-demand if they had answered “daily.” “Indifferent” and “on-demand” were coded as daily oral PrEP not preferred. Responses to the direct comparisons for daily oral and LAI PrEP were categorized as “daily,” LAI”, or “indifferent.” Participants were coded as preferring LAI PrEP to daily oral PrEP if they had answered “LAI.” “Indifferent” and “daily” were coded as LAI PrEP not preferred.

## Results

### Participant characteristics and quantitative survey responses

Participants had a median age of 27 years (interquartile range [IQR] 24–30), 35% had a college degree, median annual income was $22,000 (IQR $15,018-$27,500), and 61% identified as gay and 35% as bisexual. In the past six months, 52% reported having had condomless receptive anal sex and 62% reported having had anal sex after drinking alcohol or using drugs. Nineteen percent had moderate/high self-perceived HIV risk. Most (85%) had heard of PrEP before the study and 15% were taking PrEP at the time of the interview (median of 14 days on PrEP, IQR 5–26). Most (88%) reported being likely or very likely to take a pill everyday (Table 1).

**Table 1:** Participant characteristics (N = 26).

Characteristic	N (%)
Median age (years) (IQR)	27 (24–30)
Race	
African American/Black	21 (81)
Multiracial	5 (19)
Median annual income ($) (IQR) (n = 21)	22,000 (15,018–27,500)
Education level completed	
< College degree	17 (65)
≥ College degree	9 (35)
Sexual orientation	
Gay/Homosexual	16 (61)
Bisexual	9 (35)
Other	1 (4)
Have ever heard of PrEP	22 (85)
Reported currently taking PrEP	4 (15)
Median days taking PrEP (IQR) (n = 4)	14 (5–26)
Reported “very likely” or “likely” to take a pill everyday	23 (88)
Moderate/high perceived likelihood of ever becoming HIV infected	5 (19)
Reported having had condomless receptive sex in past 6 months (n = 25)	13 (52)
Reported having had anal sex after drinking alcohol or using recreational drugs in past 6 months	16 (62)

IQR: interquartile range; PrEP: HIV pre-exposure prophylaxis

### PrEP preference based on modality

The pill and injectable were most preferred of all the modalities (e.g., rectal gel/lubricant or enema, and patch). An oral solution and an inhalant were mentioned as other PrEP modalities that would be acceptable to use. We describe modality preferences, concerns with use, and other prominent themes that were identified.

#### Pill

Most (23/26) participants had a preference for PrEP as an oral pill when compared to other modalities. Many participants remarked that a tablet or capsule was a standard medication delivery modality was “familiar.” *“I would stick to what I'm familiar with and that would be the pill” (P44*, *22 years)*. One participant commented, *“A pill is the most convenient way*. *That is the most easy way to put things in and people are used to pills” (P13*, *27 years)*. Another participant stated, “*taking that pill is kinda easy*, *it’s convenient*, *you can always take it with you on the go*” *(P29*, *35 years)*. Being able to already take a pill daily was a factor for preferring a pill in comparison to other modalities as someone stated, *“I mean the pill is good enough for me because I know I take one pill a day” (P18*, *30 years)*.

#### Pill–Concerns with use with daily dosing

While pills were a desirable form of PrEP, eight participants noted potential challenges in adherence for daily PrEP. *“I can definitely see where it would get hard to take a pill every single day*. *It’s kind of like a birth control pill every single day like*, *oh*, *wait a minute*, *I forgot to take it for Wednesday” (P4*, *30 years)*. Along these lines, participants mentioned that non-daily schedules had some appeal. Two participants commented:

*“I think that my ideal form would be something that’s a little bit more convenient, so by convenience, I mean like, something that’s not like everyday. Even if it was like once a month or even like biweekly*. *The pill only bothers me because of the daily situation.” (P7, 24 years)*

*“Cause I wouldn’t have to like what if I*, *what if I just have one of those days where I forget, you know, to take it and then it's like, am I messed up?” (P48, 28 years)*

Some participants, in contrast, were confident they could adhere to the pill because they compared PrEP to vitamins and body-building supplements that they already take and reported their current pill-taking reminders (i.e., pillbox, phone alarm, and taking the pill with their partner). *“I know me will take uh vitamins*, *uh creatine and um I will just take it with that in the morning before I eat*. *I think it would still be best everyday” (P35*, *23 years)*. Reminders were mentioned, *“I just remember to take it everyday*, *set a [phone] alarm if I have to*. *I take vitamins everyday so it’s routine for me” (P25*, *27 years)*. Another participant reported, *“I also have a little pill reminder that I go by*. *So I put my vitamin pill*, *one vitamin pill down on that slot and okay*, *today is Wednesday*, *I’m due for a vitamin this morning” (P47*, *25 years)*. One respondent had developed a buddy system with his partner:

*“Well actually me and my partner have talked about this and pretty much we were going remind each other to make sure that we take our medicines*. *He definitely says he is going to definitely remind me to pretty much how to take it everyday like hey, good morning, don’t forget to take your medicine today, you know.” (P4, 30 years)*

#### Injection

Many (17/26) MSM found an injectable form of PrEP, regardless of frequency, preferable. Respondents compared receiving an injection to prevent HIV similar to those for other medical conditions, such as “…*I’m thinking about it like*, *like testosterone injections or insulin” (P29*, *35 years)*. Another participant reported, *“I think cause I just to me it’s just kind of like diabetes*. *They don’t like doing it but they have to do it*. *I wouldn’t do it” (P28*, *24 years)*. However, others did not want an injection without discussion of frequency of needing it as one MSM said, “*no*, *injection would be the last on my list if we’re not talking about frequency*” *(P7*, *24 years)*. Six participants mentioned that who (e.g., self or healthcare professional) administers the injection was tied to their preferences for an injectable form of PrEP. One MSM reported, *“I guess other people would be um okay with injections*. *I wouldn’t be*, *‘cause that would mean I would be doing it” (P52*, *22 years)*.

Seven participants reported fear of needles when discussing their preferences for injections versus other modalities. This fear seemed to be surmountable for some by thinking about needles as something familiar to them (i.e., a blood draw for a laboratory test) and thinking about the protection it could provide. *“I am scared of injections but it's kind of something you can’t get around especially in this day and age with the inoculation which need to get in this with you know*, *HIV testing whether it's rapid or whether it's a blood draw*. *I mean*, *you essentially get a blood draw anyway so you're getting tested for syphilis” (P57*, *28 years)*. *“I don’t like needles but I can do a needle for*, *to protect my life” (P48*, *28 years)*.

When asked about their acceptability of LAI PrEP (administered every three months), most interviewees (18/22) found LAI PrEP acceptable. Acceptability was related to familiarity with other medications that are in an injectable form, infrequent dosing, disliking injections, and knowledge of efficacy. MSM even referred to LAI PrEP as something they were familiar with, such as birth control; one states, “…*like how women take birth control*. *You know what I mean*? *Take the shot your good for three months*. *You know what I mean*?*” (P5*, *30 years)*.

Three of the seven participants who said they had a fear of or weren’t comfortable with needles were willing to use LAI due to the infrequent dosing. A participant initially reported, *“Hmm*, *not really*. *I don’t like needles*,*”* but when asked about taking a three-month injection versus a daily pill, he replied, “*yeah actually I would prefer that” (P56*, *18 years)*. One of the participants who had a fear of needles and was not willing to use LAI PrEP asked, “*Would I have to give it myself or would I have*, *would I like see a doctor and have them do it*? *I*, *well I have*, *I guess you can say I have a fear of needles so like*, *so the thought of having to do it myself*, *I'm not too sure I would you know*, *be able to have the will power to you know*, *give myself the injection” (P40*, *31 years)*.

LAI PrEP acceptability was also related to knowledge of and perceptions of efficacy. One participant was not willing to use LAI PrEP until he knew if it was “*potent enough to stay in the system*” *(P31*, *28 years)*. By contrast, another respondent found LAI PrEP acceptable because “*I believe the injection would probably get to the blood stream quicker than the pill*” *(P47*, *25 years)*.

Participants’ preferences for injections factored in a general dislike for needles, the frequency that they needed to receive an injection, and not wanting to self-administer the injection. There were few concerns with injectable PrEP. One respondent voiced a concern of pain with its use, *“Is this a painful injection*? *Yeah*, *like the level of uncomfortableness with getting this*, *is this a deep tissue injection*?*” (P55*, *24 years)*. Another participant was concerned with the timing of potential side effects with injectable PrEP that may not occur with oral PrEP pills, *“the injection might like take effect right then and there*, *you might not feel good for a couple of days because of the side effects” (P4*, *30 years)*.

#### Injection–Dosing frequency preferences

Preferred frequencies regarding injectable PrEP typically ranged from weekly to annually. One participant reported annual PrEP injections “*would be like getting the flu shot*” (P25, 27 years), while another stated, “*I think if it was an injection that you only took once a year or whatever kinda like a vaccine that would probably the best way*” *(P29*, *35 years)*. The same participant reported, “*Yeah*, *I would do a weekly injection cause I guess I’m thinking about it like*, *like testosterone injections or insulin*” *(P29*, *35 years)*. While weekly injections were not respondents’ dosing preferences, one reported, “*if it had to be every week*, *I would fit it into my schedule*. *I think it's probably something you can make into kinda like they do the diabetes where they send it to your house and you can just inject yourself with it*. *So if it is something like that*, *every week*, *that's a no brainer” (P35*, *23 years)*.

Longer dosing intervals were proposed such as *“well I'd have it once a week*, *why not once a month*?*” (P49*, *33 years)*. Some respondents thought that receiving monthly injectable PrEP was desirable, while one reported, “*Like every month*. *Yeah*, *that would be pushing it*” *(P25*, *27 years)*. Someone stated, *“I can’t say no to that*. *[A monthly injection] would be an inconvenience but if it's something like this*, *it's worth the inconvenience you know” (P41*, *23 years)*. Another reported, *“I would be willing to take it once a month*, *but ideally I’d love to take some like once every three months*, *every 90 days” (P5*, *30 years)*. Dosing intervals of longer intervals, such as three months, were preferred and familiar to MSM. *“Just like*, *like you know*, *just kinda staying the same around with other drugs you know*, *they went from having a birth control to take every day*, *now you can take it every three months you know” (P50*, *29 years)*.

#### Rectal gel/lubricant and enema

Some respondents (7/25) mentioned rectal gel/lubricant or enema as a preferred form of PrEP. Respondents who expressed views in favor of PrEP as a rectal gel/lubricant considered it easy to use with condoms and convenient to access in stores like condoms where you can potentially purchase it over the counter. *“I wouldn’t do injection*, *I guess a lube*. *Why lube*, *because I mean you have to have lube for condoms*. *I think like I could just put it on*. *I think that’d be easier*. *Instead of just putting [the pill] in my mouth every time” (P28*, *24 years)*. *“I mean a lube would be cool you just sell it [over the counter]” (P25*, *27 years)*. One person described it as “*That would be innovative*. *Yeah*. *Definitely” (P48*, *28 years)*. One person reported disliking needles so that comparatively, a lubricant would be preferred. “*I would be up for the lube*, *contrary to my piercing I am not that crazy about needles” (P57*, *28 years)*.

Enemas were not preferred over rectal gel/lubricant, nor pills or injections as PrEP. Being able to use any rectal form of PrEP was described in the context of being able to have the product with them during sex, which is not always predictable. Some participants were surprised by the suggestion, such as *“Not for me*. *I just don’t like rectal*, *rectal scares me*. *So no*. *I’m not big on that” (P22*, *30 years)*. *“Oh rectal lube and enemas out*. *Out of question for me*. *That the stuff in the butt is not that*, *no” (P52*, *22 years)*.

Adherence related to on-demand use during sex was mentioned as a factor for why rectal forms were not a preferred form of HIV prevention. One respondent mentioned, “*No*. *Because I don't always use lube*” *(P13*, *27 years)*. Another reported that he liked the concept of a rectal lube to prevent HIV but that this option would not be for everyone. *“It sounds great*. *I just know that some people sometimes don’t use lube so it sounds like a great way but it’s almost like unless you have that lube readily available all the time with you I can’t see it’s always being the most convenient thing I would say” (P29*, *35 years)*.

A rectal gel was described as an unfamiliar medication form. One MSM reported, *“Whenever you say gel I think it’s like*, *um*, *an ultrasound about how the gel is on a woman’s stomach*, *how it’s really*, *really cold*. *That’s what I think about when using a gel*. *I don’t*, *I don’t really see how that would really even be effective really when taking a certain medication*. *I don’t even know a lot of medications out there with the gels unless they’re like for rashes or something or like a lotion type*. *No*, *I don’t really think that would be necessarily a positive or optimistic thing with the* gel” *(P4*, *30 years)*. Someone else commented, *“I'm not accustomed to it*, *so I am taking other medications so for me it would definitely be the pill” (P55*, *24 years)*.

Enemas were less favored then gel/lubricants but also unfamiliar. *“Not an enema though*. *Enema sounds horrible*. *I don’t know who suggested that” (P7*, *24 years)*. Another MSM reported, *“Never done an enema” (P13*, *27 years)*.

#### Patch

Six respondents (6/25) mentioned the patch as preferred form of PrEP in comparison to other modalities. Reasons for preferring a patch were related to convenience and possibly better adherence. One participant made an analogy to the 24-hour nicotine transdermal patch for smoking cessation. *“A patch would be cool*. *You just wear it and forget it and sort of like a smoker’s patch” (P54*, *25 years)*. Another respondent reported, *“I think that my ideal form would be something that’s a little bit more convenient so by convenience*, *I mean like*, *something that’s not like every day I need to take this pill*. *I think the patch idea sounds really cool” (P7*, *24 years)*. Possible better adherence with the patch was described by a participant, *“If I have a patch*, *I will forget about the patch*, *the patch would stay on” (P13*, *27 years)*.

MSM who did not prefer to use a patch or were indifferent reported comments such as, *“No*. *I would stick to what I’m familiar with and that would be the pill*. *Definitely not anything like that [a patch]*, *anal lube or definitely not injection” (P44*, *22 years)*. *“The patch or the pill were probably be more um formidable as far as like an everyday thing” (P52*, *22 years)*. One MSM mentioned that the patch could potentially identify them as a PrEP user and could be stigmatizing. *“You know*, *you know one thing about you know*, *our community is the fact that you don’t want something that’s going on*, *that’s display ah that everyone would take notice of*. *You know*, *you’re not walking around with a big patch that’s*, *‘Oh*! *I’m HIV this or that*.*’ So I think something that’s more discrete” (P50*, *29 years)*.

#### Other PrEP modalities

Participants were asked about other PrEP modalities that they would desire, otherwise not outlined in the interview guide. Some ideas that participants came up for PrEP products included an oral solution, an inhalant, and an implantable device. *“If it was like in a liquid form that I could easily just like take while I’m drinking water” (P7*, *24 years)*. *“Ah why not an inhalant like you know*, *I mean*, *like an aspirant maybe for an inhaler just like a float or something*. *Some of the options that are there*. *Sky is the limit really when you think about it” (P49*, *33 years)*. One participant mentioned a subcutaneous implant, *“*…*like girls can literally like get something in their arm and it lasts like five years and like if there was like something Truvada or PrEPesque like that would be awesome” (P23*, *25 years)*. A participant thought an oral solution may protect oneself better from HIV and had reservations with the oral pill so he desired an oral solution. *“Like something we would rinse around and we swallow it too*, *mainly the mouth to mouth has the most cuts and already open wounds inside of it*, *swish it around inside and rinse*. *Yes*, *because I feel like if I take a pill*, *I feel like*, *I’m like just medicating myself*. *If I have an oral solution it would be just like rinsing your mouth” (P13*, *27 years)*.

### On-demand oral PrEP acceptability, concerns with use, and comparisons to daily oral PrEP

Participants were questioned about on-demand oral PrEP acceptability (i.e., their willingness to use), concerns with use, and their preference to taking an oral daily pill.

#### On-demand oral PrEP

Half of respondents (12/24) found on-demand oral PrEP acceptable. Acceptability of on-demand was related to the ability to predict sexual activity. Reasons for not willing to use on-demand involved lack of perceived efficacy. One respondent highlighted convenience with on-demand by explaining *“Cause I think it’d be easier*. *I would just*, *I’ll be more safe and I know instead of just doing it everyday*, *I can just do it right there that night and get it over it” (P28*, *24 years)*. Another MSM reported, *“No*, *the daily pill would be more inconvenient*. *The on demand would be my option” (P45*, *26 years)*.

Those who were not willing to use on-demand PrEP said it was because they could not reliably predict sex as one MSM reported, *“No*, *no*. *[I cannot predict sex]*. *And that’s yeah*, *that’s why that wouldn’t be a good idea” (P23*, *25 years)*. Another participant stated, *“Because the on demand in the heat of the moment I mean I have it with me*. *That would be*, *like that would be the reality of it*. *I guess if you carry it around your wallet or something like that but I can’t totally for my life*, *no [I would not use on demand]” (P29*, *35 years)*. One respondent mentioned, *“Because of the simple fact um our hormones*, *when people wanna have sex*, *they wanna have sex*. *I just feel like they never be thinking about a pill” (P35*, *23 years*).

Knowledge of efficacy was a factor for acceptability of on-demand PrEP for seven respondents. The concerns with efficacy were related to if on-demand PrEP would have enough time before possible HIV exposure and an adequate amount of medication in the body for it to provide protection. One respondent stated, *“it wouldn’t make me feel more protected than once daily honestly*. *But if it works then it’s definitely something I would do” (P41*, *23 years)*. Another mentioned, *“Then how would the effectiveness of that pill work if it would just be only stored in us for that moment and it wouldn’t have pre-store inside of us and when we needed it*, *we just take it after the virus has already hit probably and we are a little bit too late*. *Timing would be ineffective I feel like” (P13*, *27 years)*. One reported, *“I don’t*, *I don’t know if I would trust that on demand*. *When I say trust*, *I mean like*, *like what if it doesn’t kick in fast enough*? *Like what if there's a time window like you know*, *you have to wait like 30 minutes before and it's like I'm horny now*, *I don’t want to wait 30 minutes*. *You know*, *like I wanna do this now and then it's like*, *then it's like” (P48*, *28 years)*.

Other reasons for not favoring on-demand PrEP included how it would make MSM feel. Taking an HIV preventive method before sex may label MSM as only having condomless sex. *“A lot of people [prepare for having unprotected sex]*. *Just think about it*. *If you have PrEP as a preventative form of HIV and you could take it right before sex to prevent it then you must be having unprotected sex” (P5*, *30 years)*. Another reported, *“On-demand*, *oh that feels*, *that makes feel like I am just using it when I want to use it because I am just not caring at all” (P13*, *27 years)*.

The main concerns with potential on-demand PrEP use were medication adherence, despite being able to predict sexual activity, and affecting sexual intimacy.

Related to adherence, One participant commented about forgetting to take the pills *“in the heat of the moment” (P29*, *35 years)*. Another expressed concern that, even if people thought that they could predict sexual activity, “*they could be drinking or you know*, *they’re not thinking straight or whatever” (P24*, *34 years)*. One MSM explained, *“Um but there are also factors that go into that*. *What if you're not at home and you're just out and about and you don’t have your pills with you” (P57*, *28 years)*.

Eight respondents reported concerns with on-demand PrEP potentially affecting intimacy. One person reported, *“I mean kind of just the thought*, *it’s like*, *oh hey*, *stop I have to take this pill along with put on a condom*, *along you know*, *just the amount of things that you have to do within an intimate moment could deter that situation” (P55*, *24 years)*. Another worried about side effects during intimate moments from the pill when he mentioned, *“I would kind of like that*, *a pill before you really have sex*, *but then again it kind of stems from like if you take a pill before you have sex you don’t know how that’s gonna make you feel*, *you know*, *you might*, *you know*, *you might be tired or something or might not be able to perform like you normally do” (P4*, *30 years)*. One interviewee reported, *“people don't really want to stop what they’re doing*, *you know” (P24*, *34 years)*.

#### Daily oral versus on-demand oral PrEP

Many participants (17/24) preferred a daily oral pill to on-demand oral PrEP when asked to directly compare the two prevention strategies. Reasons for preferring a daily regimen to on-demand were comfort with taking a daily medication, an inability to predict sex, better medication adherence, not affecting intimacy, and a combination of these reasons.

MSM preferred daily oral PrEP to on-demand because the on-demand regimen was described as being tedious and unfamiliar. One MSM reported, *“No*, *I would rather take it everyday*. *Less of a hassle*. *Taking two pills before this like*, *it’s like asking me to I don’t know*. *It’s like asking me to cut the grass in the middle of my meal or something like that*. *It’s just not going to happen” (P44*, *22 years)*. Another person commented on how taking daily pills would be easier than non-daily dosing, *“I just see waking up in the morning taking it*, *having that scheduled for me works in my life” (P29*, *35 years)*.

Not being able to predict sexual activity was another reason MSM preferred the daily oral pill. *“Yeah*, *it would probably be more of an everyday preventive measure ‘cause I don’t really conspire to think about sex a lot” (P52*, *22 years)*. *“No*, *I cannot [predict sex]*. *So it’s like you know*, *that’s what I’m saying like the one pill a day it makes more logical sense to me*. *The on demand can end up being a catastrophe in my personal opinion” (P48*, *28 years)*. This was in contrast to some of the respondents who preferred on-demand PrEP because they could predict sex and dosing would be more convenient. *“That sounds better to me*. *Like you only take it only before sex*? *My lifestyle has been kind of boring recently so yeah sex is more predictable” (P7*, *24 years)*.

A few participants who preferred daily oral PrEP reported feeling safer when taking a pill everyday to protect themselves from acquiring HIV. *“I would take the pill most likely every night before I go to bed*. *I want to be safer taking it everyday than whenever I have sex” (P56*, *18 years)*. Another MSM commented, *“I think that’s smarter*. *I think it’s best to continuously take it as opposed to risking it*, *cause most people they’re not just going to do it before and after*, *I don’t think that rationale” (P31*, *28 years)*. Whereas, a participant that preferred on-demand did so because he could take it only when he would be planning to have condomless sex. *“Yeah*. *Definitely*. *Um I think personally*, *this is all personal*, *um the thing the reason I would be taken for me like before I had like okay*, *I know I’m having sex without a condom*, *I just need to go ahead and take it now” (P50*, *29 years)*.

Perceived lack of efficacy of on-demand dosing played a role in preferring daily PrEP. *“I*, *to lower of my chances [of getting HIV]*, *I would rather take it everyday*, *daily because that way I know it’s in my system” (P47*, *25 years)*. One participant reported, *“I just*, *I don’t*, *I wouldn’t feel comfortable*, *because the way I*, *I read that it works is it*, *it stays consistent in your blood stream if you take it once daily and not*, *maybe you had a full meal*, *it prevents your body from absorbing” (P41*, *23 years)*. Another stated, *“I feel like right before wouldn’t be effective enough” (P35*, *23 years)*.

Two people changed their minds during the interview from initially preferring on-demand to then preferring daily PrEP. *“It's pretty sporadic so in a sense*, *you know*, *actually can I take that back*, *just thinking about that just that scenario*, *it would probably be more convenient to do the everyday because I*, *it's not usually planned*, *the sexual activity” (P46*, *28 years)*.

### Daily oral versus long-acting injection PrEP

Most participants (15/21) preferred LAI compared to the daily oral pill. Reasons MSM chose LAI over a daily pill included dislike for taking pills and potentially better medication adherence.

Related to medication adherence, MSM who preferred LAI reported comments such as, *“Cause I wouldn’t have to like what if I*, *what if I just have one of those days where I forget*, *you know*, *to take it and then it's like*, *am I messed up*? *You know*, *but I never heard of the three-month injection until today you know*. *So if I had to choose between an injection or the pill*, *I would do the injection” (P48*, *28 years)*. Another reported, *“I would*. *It's just in case you forget your pill*. *You know*, *I forgot to take the pill or you know*. *It would just be easier to just go and get a three month injection” (P45*, *26 years)*. One participant commented that the injectable could help people if they have poor adherence related to psychosocial stressors. *“Um the pill actually is a good idea but taking it everyday uh it can be a little stressful because you known um people always uh no telling what’s going on with someone’s life where they can be stressed out*, *so they probably don’t even get out of bed in the morning so they don't take the pill*. *So I feel something like a shot would be more better*, *like coming in probably every three months as they do they checkup and just get the shot*. *Yeah*. *I feel like that would be something more better that I wanna do” (P35*, *23 years)*.

Some participants preferred LAI PrEP due to their dislike for taking pills. One person mentioned, *“Probably a three month injection cause pills are not fun” (P46*, *28 years)*. While another reported, *“A pill is acceptable to me*. *An injection that you got once every three months would be a lot better*. *Other than that*, *I don’t think I would take any other medication any other way” (P41*, *23 years)*.

Reasons for preferring daily oral pills as PrEP included a general dislike for injections as one MSM reported, *“Shots are not that painful but still the thought of a regular shot is kinda um (laughs)*. *I don’t want to do that but it really isn’t that much*, *it really isn’t that much of an inconvenience to take a daily pill” (P54*, *25 years)*. Another participant stated, *“I don’t wanna be injected with anything*. *I don’t like shots*. *I don’t like needles*. *I would prefer to stay away from those*. *I’d rather swallow a pill” (P44*, *22 years)*. Of the seven participants who had reported a fear of needles, three preferred daily oral PrEP due to this reason.

A few participants who preferred daily oral PrEP did so because they did not know LAI PrEP’s efficacy. *“How long does it take for it to kick in*? *I would if I knew its effectiveness*. *If I knew how effective it would be over these three months*, *then possibly yes” (P55*, *24 years)*. Another MSM commented, *“If potent enough to stay in the system*, *yes” (P31*, *28 years)*.

Only one participant was indifferent to either PrEP form, *“The pill or a three-month injection will work fine for me*. *Either one of those two work great for me” (P47*, *25 years)*.

## Discussion

This study explored biomedical HIV prevention preferences for young adult African American MSM, a population that is among the highest risk for HIV [1, 2]. While most participants preferred oral and injectable PrEP, about a quarter each preferred rectal or patch forms of PrEP. Participants reported aversion to needles when discussing injectable PrEP, including LAI, but also suggested these challenges could be addressed. Some participants questioned the ability to maintain therapeutic levels of protection with non-daily dosing, but on-demand oral and LAI as PrEP were acceptable to many men in the study. Concerns with on-demand PrEP use included the ability to predict sexual activity and adhere to the dosing requirements. Most participants preferred LAI to a daily pill, and the daily pill to an on-demand pill.

Our findings provide further support that LAI PrEP was highly accepted, and preferred to oral daily PrEP by young African American MSM [33–35, 47]. We identified that a fear of needles may impede LAI use in some, but these fears were assuaged through having a health provider administer the injection, thinking of needles as a blood draw that they are familiar with, or remembering that the injection will provide protection from HIV. Educational programing that addresses these reported concerns with injectable PrEP use and that incorporates these case scenarios (i.e., injectable options for birth control and diabetes treatment) is crucial for increasing PrEP uptake by community members during early phases of the PrEP implementation process with these new products [32, 52]. Furthermore, injectable PrEP could be administered by a pharmacist or other health provider within pharmacy-based clinics, an emerging delivery model in the US, to address some of these reported barriers [53, 54].

The most preferred injectable PrEP frequency reported in this sample was every three months, but some participants would accept receiving injectable PrEP on monthly or weekly intervals. According to most participants, a LAI every three months was preferred over receiving a daily pill. Similar to our study, Meyers et al. found LAI PrEP to be acceptable (80.7%) among MSM, defined as willing to use, and it was preferred, as a three-month injection, over a daily pill (79.2%) [33]. Parsons et al. demonstrated that 53.6% of gay and bisexual men wanted a LAI every three months [34]. However, other studies found 21.7% gay and bisexual men preferred the modality (i.e., pill or injectable) that was more effective, while only 5.7% of African American gay and bisexual men preferred the LAI [34]. John et al. reported LAI was acceptable by 30.8% of the study participants, but that 34.6% would also choose the most effective product if they had to choose between daily oral and LAI PrEP [35].

Intermittent on-demand PrEP is recommended by the European AIDS Clinical Society and French, Australian, and Canadian ministry of health guidelines for MSM, but is not a widely accepted alternative for daily PrEP [27, 38, 40–43, 55, 56]. The CDC and WHO have not recommended on-demand dosing, however obtaining preferences regarding this option is crucial as the toolbox of different biomedical HIV prevention options is created [15, 39]. Many African American MSM in our study preferred taking a daily pill to on-demand, in contrast to other studies [44, 47]. Reasons for the preference of daily PrEP to on-demand cited by MSM in the study were MSM were already accustomed to taking daily medications, had medication adherence support systems in place (i.e., pillbox, phone alarm, and taking the pill with their partner), and the inability to predict sexual activity. In this sample, the majority (23/26) of MSM also felt they were “very likely” or “likely” to take a pill everyday, while more than half (16/26) reported having anal sex under the influence of alcohol or drugs within the past six months. Grant et al. cited poor adherence among African American MSM when PrEP was taken non-daily [20].

Other factors that played a role with acceptability and concerns with on-demand use were lack of knowledge of efficacy and affecting sexual intimacy. Other studies similarly report the ability to accurately plan or predict sex by MSM affect their willingness to use on-demand PrEP or episodic PrEP (daily oral PrEP taken over discrete episodes of highest risk, such as vacations) [26, 44, 47]. Among those who receive prescriptions for daily versus on-demand PrEP, Greenwald et al. demonstrated that younger MSM (mean 36.7 years); compared to older patients (mean 39.2 years), were more likely to receive daily PrEP versus on-demand within a sample of 1073 MSM prescribed PrEP in Canada [57]. The decision for daily PrEP prescribing was associated with behavior risk profiles reported by MSM, such as having spontaneous sex with multiple partners [57].

About half (12/25) of the participants mentioned a rectal and/or patch modality as a preferred form of PrEP. These two modalities are not commonly explored in the PrEP literature. Kubicek et al. reported that young MSM made the connection of rectal microbicides with lubricants and that, in the setting of unknown efficacy or side effects, they were more receptive to using rectal microbicides compared to daily oral PrEP [58]. In contrast, Carballo-Diéguez, et al. found that, MSM and transgender women preferred daily oral PrEP (73%) to a rectal gel applied before and after receptive anal intercourse (19%) when participants were asked which modality they liked the most [25]. In another study of MSM recruited online, participants were less likely to use any of four rectal modalities (i.e., gel or suppository used before or after intercourse) versus daily oral PrEP [47]. In terms of preferring a patch, participants considered this preventive measure similar to using a Nicotine patch for tobacco cessation, but potential stigma associated with being able to easily identify PrEP users. Currently, a thin-film polymer subcutaneous implant has been developed to help address user compliance and therapeutic effectiveness, which was found to deliver antiretroviral medication for HIV prevention up to 90 days in clinical trials [21]. Rectal forms of PrEP under investigation include tenofovir-based gels, enemas, and douches [59, 60, 61]; trials involving rectal gels have demonstrated safety, tolerability, and high levels of use among MSM [61, 62]. Further studies should assess the acceptability and barriers to use of rectal and patch forms of PrEP to anticipate product uptake as development occurs. The results of this and other studies can inform the conceptualization and implementation of new PrEP products tailored to meet young African American MSM’s preferences.

Along with PrEP product education programs during future implementation, program content will need to address the low self-perceived risk for HIV among young MSM identified in this sample (81%) and supported by other studies [63, 64]. Low self-perceived risk may have affected the product preference choices and the willingness to use products. Perceived risk was low high prevalence of condomless anal sex and anal sex under the influence of drugs or alcohol, as well as high population-specific HIV incidence rates. Low self-perceived risk is associated with low PrEP initiation and will serve as a definitive barrier to any new PrEP product uptake [63, 64].

Study limitations include a small sample size and single geographic site that limit generalizability. Some modalities were not included in this study (e.g., rectal douches). The study asked participants to consider theoretical PrEP options without exact efficacy estimates, and exact dosing frequencies for on-demand and LAI; therefore, participants may have had difficulty conceptualizing the modalities. Furthermore, the sample contained a large proportion of persons who were not on the currently available daily oral PrEP, which further limits the ability for participants to conceptualize theoretical PrEP options.

## Conclusions

Young African American MSM are disproportionately at higher risk for HIV and, thus, tailoring PrEP product development and implementation for this population should be a national priority [1, 2]. We identified preferable forms of PrEP and the potential challenges to successful implementation across a marginalized population. Study findings should be incorporated into PrEP product development strategies among manufacturers and other stakeholders, and inform future implementation efforts to maximize uptake and adherence among populations at-risk for HIV.

## Supporting information

S1 File Qualitative interview guide. (DOCX)
