# Supplementary material for: Pre-exposure prophylaxis for HIV prevention preferences among young adult African American men who have sex with men
Source: PLoS One. 2018 Dec 28;13(12):e0209484. doi: 10.1371/journal.pone.0209484 (PMC6310267; doi:10.1371/journal.pone.0209484)
Supplement: S1 File — (DOCX) [file pone.0209484.s001.docx]

**PrEP In-Depth Semi-Structured Interview Guide**

**PrEP Awareness**

Pre-exposure prophylaxis or PrEP is an HIV medication you can take BEFORE being exposed to HIV and it can lower your chances of getting HIV. It has been shown to decrease your chance of getting HIV by more than 90% if it is taken everyday. Right now, a medication called Truvada is used for PrEP and it is one pill taken daily.

1. Have you heard about HIV pre-exposure prophylaxis (PrEP) before?

- *Probe:* Where?

1. What do you think about daily oral PrEP?

- *Probes:* Is it a good idea? Why or why not? Are there any concerns?

1. Would you be willing to use daily oral PrEP?

- *Probe:* Why or why not?

**PrEP Preferences**

1. If there could be different ways to protect yourself from HIV using PrEP, which way would you prefer from a list of a pill, an injection, rectal gel, lubricant or enema, skin patch, and/or other?

- *Probe:* Why?

1. There are studies that show that an injection could be given every three months for PrEP in the future. How do you feel about this?

- *Probe:* Are there any concerns?

Would you be willing to use this?

- *Probe:* Why or why not?

Would you prefer taking a daily oral pill or the injection if it was available?

- *Probe:* Why?

1. There are studies that show that using 2 pills before sex and 2 pills after sex (also called on-demand PrEP) may work. How do you feel about this?

- *Probe:* Are there any concerns?

Would you be willing to use this?

- *Probe:* Why or why not?

Would you prefer taking a daily oral pill or on-demand PrEP if it was available?

- *Probe:* Why?

1. When is the preferred time to take PrEP for you regardless if it is a pill, injection, skin patch, or other? Would it be daily, every week, every month, every three months, and/or other times?

- *Probe:* Why?

Any additional thoughts?

Thank you for your input.
